# Derivation and Validation of the Cognitive Impairment Prediction Model in Older Adults: A National Cohort Study

**DOI:** 10.3389/fnagi.2022.755005

**Published:** 2022-03-04

**Authors:** Mingyue Hu, Yinyan Gao, Timothy C. Y. Kwok, Zhanfang Shao, Lily Dongxia Xiao, Hui Feng

**Affiliations:** ^1^Xiangya School of Nursing, Central South University, Changsha, China; ^2^Xiangya School of Public Health, Central South University, Changsha, China; ^3^JC School of Public Health and Primary Care, The Chinese University of Hong Kong, Hong Kong, Hong Kong SAR, China; ^4^College of Nursing and Health Sciences, Flinders University, Adelaide, SA, Australia; ^5^Oceanwide Health Management Institute, Central South University, Changsha, China; ^6^National Clinical Research Center for Geriatric Disorders, Xiangya Hospital, Central South University, Changsha, China

**Keywords:** prediction model, cognitive impairment, modifiable risk factors, aging, older adults

## Abstract

**Objective:**

This prediction model quantifies the risk of cognitive impairment. This aim of this study was to develop and validate a prediction model to calculate the 6-year risk of cognitive impairment.

**Methods:**

Participants from the Chinese Longitudinal Healthy Longevity Survey (CLHLS) 2008–2014 and 2011–2018 surveys were included for developing the cognitive impairment prediction model. The least absolute shrinkage and selection operator, clinical knowledge, and previous experience were performed to select predictors. The Cox proportional hazard model and Fine-Gray analysis adjusting for death were conducted to construct the model. The discriminative ability was measured using C-statistics. The model was evaluated externally using the temporal validation method *via* the CLHLS 2002–2008 survey. A nomogram was conducted to enhance the practical use. The population attributable fraction was calculated.

**Results:**

A total of 10,053 older adults were included for model development. During a median of 5.68 years, 1,750 (17.4%) participants experienced cognitive impairment. Eight easy-to-obtain predictors were used to develop the model. The overall proportion of death was 43.3%. The effect of age on cognitive impairment reduced after adjusting the competing risk of death. The Cox and Fine–Gray models showed a similar discriminative ability, with average C-statistics of 0.71 and 0.69 in development and external validation datasets, respectively. The model performed better in younger older adults (65–74 years). The proportion of 6-year cognitive impairment due to modifiable risk factors was 47.7%.

**Conclusion:**

This model could be used to identify older adults aged 65 years and above at high risk of cognitive impairment and initiate timely interventions on modifiable factors to prevent nearly half of dementia.

## Introduction

There are more than 50 million people living with dementia globally, with a figure increasing to 152 million by 2050 Alzheimer’s Disease International [ADI] (2019). China has the largest population with dementia (9.5 million), followed by the United States (4.2 million) Alzheimer’s Disease International [ADI] (2015). The annual cost associated with dementia is estimated at $1.33 trillion in 2020, with a figure rising to $9.12 trillion in 2050 (Jia et al., 2020). There is no cure for dementia, but the Lancet Commission recently reported that the number of dementia could be reduced by 40% by targeting 12 modifiable risk factors (Livingston et al., 2020). The first step is screening out people at high risk of dementia, but screening for every older adult is not necessary and possible because of the heavy burden and ethical issues. Therefore, an easy-to-use prediction model for health professionals in various care settings is needed to roll out those with low-risk and select high-risk dementia for further management.

There are easy-to-use (i.e., without expensive or unavailable biomarkers) and nationally representative dementia prediction models to assess the risk of dementia in care settings quantitatively, but there might be shortcomings leading to distorted estimates of the predictive performances of the model (Li et al., 2018; Hou et al., 2019; Licher et al., 2019; Zhou et al., 2020). One important consideration is related to the effect of death. In one of the studies (Li et al., 2018) that used the Framingham Heart Study to develop a 5-, 10-, and 20-year dementia risk prediction model, the area under the curve (AUC) was 0.72 with predictors of demographic characteristics and diseases. Two studies (Zhou et al., 2020; Hu et al., 2021) used the Chinese Longitudinal Healthy Longevity Survey (CLHLS) to develop a cognitive impairment prediction model, with an average AUC of 0.80. However, these studies did not consider death, a critical competing risk of dementia. Given that dementia generally occurs in late life, failure to account for such an important competing risk could result in bias, limiting the practical use of current models (Fine and Gray, 1999).

Another consideration is related to incorporation bias. Some dementia prediction models (Licher et al., 2019; Hu et al., 2021) included predictors that are part of the assessment of dementia, such as the cognition score or physical ability, resulting in optimistic estimates of the model performances. The last consideration involved the generalization of the prediction model to different ethnic groups. Licher et al. (2019) developed a dementia model with the AUCs of 0.78 of the basic model and 0.86 of the augmenting one (such as cognition and genetic predictors) while adjusting for the effect of competing risk of dementia. However, this model was developed in Caucasian descent, and it was doubtful whether the predictors and model were applicable to Asians. To fill these gaps, we aimed to develop and validate the cognitive impairment prediction model that would take account of the competing risk of death and exclude predictors of the definition of dementia in Chinese people.

## Materials and Methods

The Transparent Reporting of a Multivariable Prediction Model for Individual Prognosis or Diagnosis (TRIPOD) was followed (Collins et al., 2015; Supplementary Table 5).

### Participants

Participants were chosen from the CLHLS, one of the largest national longitudinal studies for investigating the health of older Chinese adults. The sampling frame covered about 85% of the total population of China^1^. In this study, we selected two cohorts of participants (2008–2011 and 2011–2018) for model development. Individuals were included if they (i) were aged 65 years and above; (ii) resided in their homes, retirement villages, and all types of aged care institutions; (iii) were normally cognitive with Chinese Mini-Mental State Examination (cMMSE) scores of 18 years and above at baseline (Hu et al., 2021); and (iv) had at least one follow-up. Individuals were excluded if they had serious diseases, including dementia and cancer.

We used a separate cohort of CLHLS from 2002 to 2008 for external validation. The inclusion and exclusion criteria were identical to the cohort above in the validation cohort.

### Candidate Predictors

The selection of predictors was based on clinical importance, scientific knowledge, and predictors identified in previously published studies and data available in the database (Cooper et al., 2015; Livingston et al., 2020; Hu et al., 2021). Several candidate predictors were chosen, including demographic characteristics, lifestyles, diseases, physical function, and mood. The detailed information is shown in Supplementary Table 1.

### Assessment of Cognitive Impairment

The cognitive function was assessed by the cMMSE (Zhang et al., 2006), which was culturally translated from the international standard of the MMSE questionnaire. It consists of 24 items with six dimensions, namely, orientation, registration, naming, attention, calculation, recall, and language, with higher scores indicating better cognitive function. The cMMSE has been validated among the Chinese elderly population. It has shown that the cMMSE is not affected by the educational level (Gao et al., 2015). Consistent with previous studies, a score of 18 has been set out as a cognitive impairment threshold, that is, an individual with a score below the threshold is considered impaired (Zhang et al., 2006; Hu et al., 2021). In the survival analysis, participants were censored at the date the cognitive impairment was confirmed (cMMSE scores 17 or less), or death, or the year the follow-up ended (Jagger et al., 2015; Hou et al., 2018).

### Statistical Analysis

Continuous variables were reported as medians with interquartile ranges (IQRs). The category variables were presented as numbers with proportions.

There were four steps in the data handling process: missing data, outliers, predictor selection, and nonlinear association with outcomes. First, a small percentage of the data of most predictors was missing (<3%), except for the drinking index (9.4%) and smoking index (13.0%). The multiple imputation was conducted with numbers of multiple imputation of 5. Second, to prevent the extreme effects of predictors, outliers below the bottom 1% and above the top 99% were replaced with 0.01 and 0.99 percentile values, respectively (Licher et al., 2019).

Third, predictors selection was conducted based on the Prediction model Risk of Bias Assessment Tool (PROBAST) statement (Moons et al., 2019). Notably, predictors selection did not reply on the univariate analysis that could introduce bias (Moons et al., 2019). To effectively select candidate predictors, we selected predictors based on our previous experience (Hu et al., 2021) and previously published studies (Hou et al., 2019; Licher et al., 2019; Livingston et al., 2020; Zhou et al., 2020). In addition, to avoid model overfitting and model misspecification, the least absolute shrinkage and selection operator (LASSO) with 10-fold cross-validation was employed (Pavlou et al., 2015).

Fourth, to dichotomize continuous predictors, the points were used if they had predefined cut points. For those without predefined points, the non-linear or linear associations between the predictors and the outcome were examined using restricted cubic splines to avoid information loss and maintain simplicity, thus aiding clinical interpretation (Moons et al., 2019).

In the statistical analysis, we developed a 6-year prediction model using two methods, namely, the Cox proportional hazard model and Fine–Gray analysis. The latter allowed to take account for death when developing the cognitive impairment prediction model. Data were expressed as the hazard ratio with 95% confidence intervals. The nomogram was constructed to vividly present the outcome and improve practical use in preventing cognitive impairment. The population attributable fraction (PAF) was calculated using the following formula^2^ :


$$P\,A\,F=\frac{\sum_{i=1}^{n}P_{i}\,R\,R_{i}-\sum_{i=1}^{n}P_{i}^{\prime }\,R\,R_{i}}{\sum_{i=1}^{n}P_{i}\,R\,R_{i}}$$

where *P*_*i*_ is the proportion of population at exposure level *i*, current exposure; *P’_*i*_* is the counterfactual or ideal level of exposure; *RR*_*i*_ is the risk ratio at exposure level *i*; and *n* is the number of exposure levels.

The hazard ratio was considered equal to the risk ratio, given the low incidence (Zhang and Yu, 1998). All statistical analyses were performed using the R version 4.0.4 software with major packages of survival, survminer, riskRegression, averisk, and regplot (Park et al., 2021).

### Internal Validation and External Validation

To examine the internal validation, we used the cross-validation with the 10-fold sampling method to examine the robustness of the prediction model and found consistent results in selection steps using the LASSO technique. To check the external validation, we performed the temporal validation (Collins et al., 2015) using the cohort of 2002–2008. In both internal and external validations, we quantified the discriminative ability of models using the C-statistics for survival data (Licher et al., 2019). Given the influence of age on the prediction model, we used a model based on age alone as a reference for analyses.

### Stratified Analysis

Stratified analyses were carried out based on age, sex group, and levels of cognitive function at baseline. The effect of loss to follow-up on the outcome was analyzed by treating the individuals lost to follow-up as censoring, impairment, and death separately.

The CLHLS study was approved by the research ethics committees of Duke University and Peking University (IRB00001052-13074).

## Results

### Study Population

**FIGURE 1:**
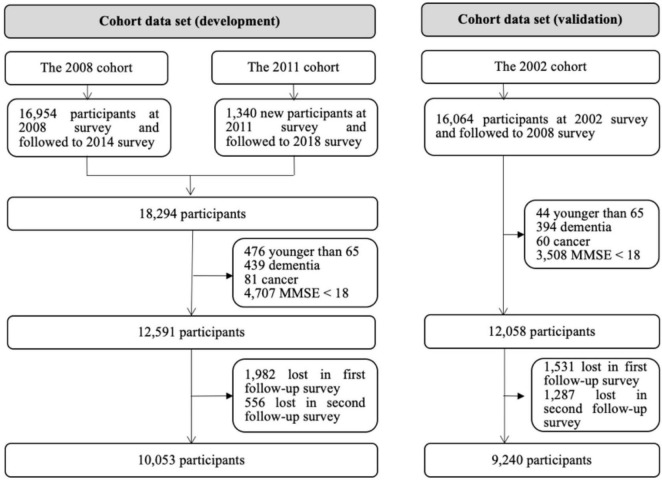
Flowchart of the study population.

In the cohort data set for the development of the prediction model, a total of 10,053 participants were included. During a median follow-up period of 5.68 years (IQR: 3.87–5.92 years), a total of 1,750 impairments (17.4%) were observed, representing 34.8 impairments per 1,000 person-years. In the cohort data set for external validation of the prediction model, a total of 9,240 participants were included in the final analysis. The detailed flowchart of the study population is shown in Figure 1.

### Predictors Selected

The detailed selection process by LASSO is shown in Supplementary Figure 1 and Supplementary Table 2. We then used the multivariate regression analyses through the Cox proportional hazard model to further select predictors. We finally chose eight predictors (i.e., age, sex, education, marital status, activity duration, playing cards or mah-jongg, watching TV or listening to the radio, and presence of stroke or cardiovascular diseases) to develop the model. Age was dichotomized into four categories (i.e., 65–74, 75–84, 85–94, and 95 years and above), and the activity duration was categorized into four groups (i.e., 0, 1–15, 16–40, and 41 years and above) based on the results of the restricted cubic spline. The detailed nonlinear associations between two predictors and cognitive impairment are shown in Supplementary Figures 2, 3. The detailed information on baseline characteristics is shown in Supplementary Table 3.

**FIGURE 2:**
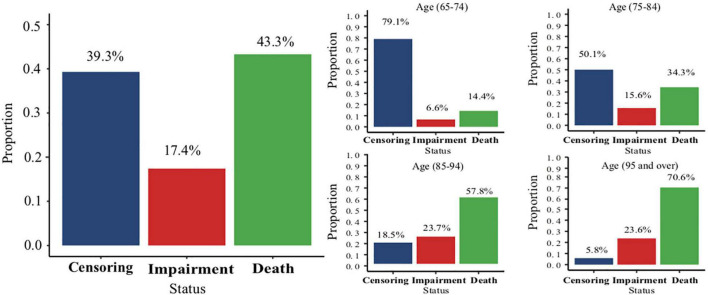
The overall proportion of different status and the proportions of different status in each age group.

At the end of the follow-up, the overall proportions of impairment and death were 17.4 and 43.3%, respectively. The proportion of death in the 95 years and above age group was nearly five times more than that in the 65–74 years age group (Figure 2). The effect of age on the cognitive impairment was relatively reduced when using the Fine-Gray analysis (HR range: 2.1–3.02), compared with the Cox proportional hazard model (HR range: 2.46–4.52). The detailed information is shown in Supplementary Table 4.

### Model Development and Validation

The Cox proportional hazard analysis and Fine–Gray analysis showed similar results. In the development dataset, the C-statistics was 0.71 (95% CI 0.70–0.73) in the Cox proportional hazard analysis. In the validation dataset, the model showed a slightly attenuated discriminative ability with a C-statistics of 0.69 (95% CI 0.65–0.69) in the Cox proportional hazard analysis. The differences were significant when comparing the full model with the model of age alone (*p* < 0.00). The detailed information is shown in Table 1.

**TABLE 1:** Discriminative ability for the cognitive impairment prediction model in both development and validation datasets with a model based on age alone as reference.

Data sets		Age alone		Full model		Model comparison (*p*-value)*
			
		C-statistics	95% CI	C-statistics	95% CI	
Development dataset	Cox proportional hazard analysis	0.67	0.66–0.68	0.71	0.70–0.72	<0.00
	Fine–Gray analysis	0.65	0.64–0.67	0.71	0.70–0.73	<0.00
Validation dataset	Cox proportional hazard analysis	0.65	0.64–0.66	0.69	0.67–0.70	<0.00
	Fine–Gray analysis	0.63	0.61–0.64	0.67	0.65–0.69	<0.00

*Model comparison used chi-square test in the Cox proportional hazard analysis and Delong test in the Fine–Gray analysis.

### Stratified Analysis

The prediction model performed relatively better in older individuals with higher MMSE scores. In individuals aged 65–74 years, the model showed a higher discriminative ability. The detailed information on the stratified analysis is shown in Table 2.

**TABLE 2:** Stratified analyses of the discriminative ability for the cognitive impairment prediction models in the development data set.

Sensitivity analyses	Incidence rate (n/N)	Cox proportional hazard analysis		Fine–Gray analysis	
			
		C-statistics	95% CI	C-statistics	95% CI
**Cognitive function at baseline**					
MMSE score of 26-30	14.5% (961/6645)	0.73	0.71–0.75	0.74	0.72–0.76
MMSE score of 18-25	23.2% (789/3408)	0.63	0.61–0.65	0.63	0.60–0.65
Age					
65–74	6.6% (159/2411)	0.66	0.62–0.71	0.69	0.62–0.75
75–84	15.6% (420/2693)	0.62	0.59–0.65	0.65	0.61–0.68
85–94	23.7%(763/3220)	0.62	0.59–0.64	0.63	0.60–0.65
95 and over	23.6%(408/1729)	0.61	0.58–0.64	0.61	0.58–0.64
**Loss to follow-up**					
Censoring	13.9% (1750/12591)	0.71	0.70–0.72	0.71	0.68–0.74
Impairment	34.1% (4288/12591)	0.63	0.62–0.63	0.62	0.61–0.63
Death	13.9% (1750/12591)	–	–	0.71	0.70–0.72
**Sex**					
Male	17.4% (864/4970)	0.71	0.69–0.72	0.69	0.66–0.71
Female	17.4%(886/5083)	0.70	0.68–0.72	0.67	0.67–0.65

*MMSE, Mini-Mental State Examination.*

### Practice Use of Cognitive Impairment Prediction Model

The nomogram based on the Cox proportional hazard analysis vividly illustrated the contribution of each predictor to the cognitive impairment. It allowed drawing a straight line down to determine the estimated probability of cognitive impairment more than 6 years (Supplementary Figure 4).

The sensitivity, specificity, positive predictive value, and negative predictive value at different cutoff points to predict the 6-year risk of cognitive impairment are shown in Table 3. Given the cutoff point of 0.10, this model could identify 74% of participants who would develop cognitive impairment and 43% of participants who were not likely to develop cognitive impairment 6 years later.

**TABLE 3:** Sensitivity, specificity, positive predictive value (PPV), and negative predictive value (NPV) at different cutoff points to predict 6-year risk of cognitive impairment in the development dataset.

Cutoff point	Sensitivity	Specificity	PV–	PV+
0.70	0%	100%	–	–
0.03	100%	0%	–	–
0.10	74%	43%	90%	21%
0.15	57%	59%	87%	23%
0.20	42%	72%	86%	24%

*PV−, negative predictive value; PV+, positive predictive value.*

### Population Attributable Fraction

The results of PAFs showed that the proportion of 6-year cognitive impairment associated with these eight factors was 80.9%. Targeting four modifiable predictors, namely, activity duration, playing cards or mah-jongg, watching TV or listening to the radio, and stroke/cardiovascular diseases would cause a decrease of 47.7% of 6-year cognitive impairment.

## Discussion

We developed a model to predict cognitive impairment with a follow-up duration of up to 6.6 years among older adults aged 65 years and above. The model included eight predictors (i.e., age, sex, education, marital status, activity duration, playing cards or mah-jongg, watching TV or listening to the radio, and stroke and cardiovascular diseases) with a good discriminative ability. This model could help practitioners and nurses in various care settings identify older adults at high risk of 6-year cognitive impairment and thus give them continuing monitoring and interventions.

Predictors in this model included age (Kivipelto et al., 2006; Anstey et al., 2013; Barnes et al., 2014; Walters et al., 2016), sex (Kivipelto et al., 2006; Anstey et al., 2013; Walters et al., 2016), education (Kivipelto et al., 2006; Anstey et al., 2013; Barnes et al., 2014), and stroke (Anstey et al., 2013; Barnes et al., 2014; Walters et al., 2016), which were also included in Licher et al. (2018) the Cardiovascular Risk Factors, Aging, and Dementia (CAIDE) from Finland (Kivipelto et al., 2006), Brief Dementia Screening Indicator (BDSI) from America (Barnes et al., 2014), Australian National University Alzheimer’s Disease Risk Index (ANU-ADRI) from Australia (Anstey et al., 2013), and Dementia Risk Score (DRS) from England (Walters et al., 2016).

The incidence rate of cognitive impairment in our study was similar to previous studies. Compared with previous studies that only selected older people living in their own homes, the selection criteria of participants did not contribute to a relatively higher percentage of cognitive impairment identified in our study. The 6-year incidence rate of cognitive impairment was ranged from 5.6 to 9.4% (compared with 6.6% in our study) among older adults aged 65–74 years in the Cardiovascular Health Study (Barnes et al., 2014). The 6-year incidence rate of the cognitive impairment was ranged from 15.2 to 26.8% (compared with 15.6% in our study) in the Sacramento Area Latino Study on Aging (Barnes et al., 2014). The reason might be that a higher proportion (98.6%) of older adults were living in their own homes in our study.

Our model showed that being single increased the risk of dementia compared with married individuals. This finding was consistent with previous studies. The previous meta-analysis (Sommerlad et al., 2018) and our experience (Hu et al., 2021) have suggested that being widowed or single could increase the risk of dementia compared with married individuals. One reason behind this relation might be due to dyadic coping (Falconier and Kuhn, 2019). Another reason might be that married people may be more likely to participate in physical and social activities conducive to cognitive reserve (Hu et al., 2021). However, this strong predictor has been overlooked in previous studies (Kivipelto et al., 2006; Anstey et al., 2013; Barnes et al., 2014; Walters et al., 2016). Further studies should examine the association between the marital status and cognitive impairment.

The activity duration was selected as an important predictor of future cognitive impairment. Physical activity could improve the hippocampal volume, induce neurogenesis, and generate more neurotrophic factors (Mariotti et al., 2014; Urbán and Guillemot, 2014). However, only a long period of activity was predictive of the impairment. There might be a dose-response relationship between physical activity and cognition. The dose could be determined by different intensities, durations, and types (Tolppanen et al., 2015; Palta et al., 2019). One longitudinal study (Palta et al., 2019) with 10,705 participants and a follow-up of 17.4 years showed that moderate and high-intensity physical activities were associated with low incidences of cognitive decline but not the low intensity. Another study (Tolppanen et al., 2015) followed more than 3,000 middle-aged and older adults up to 36 years, and results showed that, after midlife, maintaining high physical activity or increasing physical activity was associated with lower dementia risk.

Two mental stimulation activities, playing cards and mah-jongg and watching TV or listening to the radio, were predictors. These two cognitive activities were called novel and passive cognitive activities, respectively (Carlson et al., 2008). The difference between these two cognitive activities is that the former needs a response. Several studies have investigated the association between these two types of cognitive activity and cognitive impairment. One study (Carlson et al., 2008) in male twins controlling for genetic and environmental factors showed that the midlife cognitive activity was related to a 26% risk reduction for dementia, particularly among twin pairs at an elevated genetic risk. Another recent study (Najar et al., 2019) that followed 800 women with a median age of 44 years exhibited a similar result. This phenomenon could be explained by the cognitive reserve hypothesis of the brain and “use it or lose it” theory. Brain reserve indicates that individuals with a greater brain reserve require higher levels of change in cortical thickness, β-amyloid, and regional atrophy to exhibit clinical symptoms of cognitive impairment (Tucker and Stern, 2011; Najar et al., 2019). The “use it or lose it” theory means that disuse could lead to subsequent atrophy of cognitive skills (Stern and Munn, 2010). Future detailed information on the type, duration, intensity, and timing of that activity might improve the accuracy of the model (Stern and Munn, 2010; Sajeev et al., 2016).

Consistent with previous studies, our model performed better in those younger older adults aged 65–74 years and those with higher MMSE scores of 26–30. One of the studies (Licher et al., 2018) included 2,710 older adults with a mean age of 71.2 years, where the general discriminative ability of the dementia prediction model was 0.79, but the C-statistic decreased to 0.57 among individuals older than 80 years. The study by Hall et al. (2019) constructed the dementia prediction model among older adults aged 85 years and above, and results showed that the predictors were different from those in previous reports of the younger population. Predictors of age, sex, and vascular and lifestyle factors no longer had the predictive ability. Objective predictors, such as total brain volume and hippocampal volume, helped predict cognitive impairment in the oldest-old (Licher et al., 2018). More promising blood-based predictors such as the plasma total tau level and lipids with high accuracy of 90% have been explored (Mapstone et al., 2014; Pase et al., 2019). Their usability in various care settings deserves attention.

In 2020, the US Preventive Services Task Force (USPSTF) issued that the balance of benefits and harms of screening for cognitive impairment was not determined due to insufficient evidence, making the role of our prediction model controversial. On one hand, early identification allowed early interventions. The USPSTF recommended that early signs or symptoms of cognitive impairment should be noticed and evaluated as appropriate because it allowed for identification and treatment of reversible causes and more involvement from patients and families. One study (Ngandu et al., 2015) involving 2,654 individuals investigated the effect of a 2-year multidomain intervention on cognitive function in at-risk older adults, and results showed that cognitive function was improved or maintained. In contrast, even though the harm of screening is limited, potential harms should be addressed, such as ineffectiveness and adverse effects of pharmacological interventions, potential stress, and lower quality of life caused by the awareness of a diagnosis of cognitive impairment (Stites et al., 2017). However, the main aim of our study was related to early prevention and did not emphasize diagnosis, and thus harms due to diagnosis were not applicable. Theoretically, 47.7% of dementias could be prevented or delayed through early detection and interventions on modifiable risk factors. This number was similar to that published by the Lancet Commission that emphasized the importance of early intervention. Due to the relatively low incidence of cognitive impairment, the positive predictive value was low, whereas the negative predictive value was high. Nevertheless, since this model aims to screen out individuals at high risk of cognitive impairment for further monitoring and non-pharmacological interventions, this model could meet this demand.

### Strengths and Limitations

Our study had some limitations. For instance, we used older data from 2008 to 2011 to develop the model. Therefore, the effect of some predictors on cognitive impairment today might be slightly different due to the changing distribution of predictors, such as the increase of prevalence of diabetes, which has an impact on confidence intervals of risk factors. However, as confidence intervals of the risk factor were determined by three factors (University of Connecticut, 2021), namely, sample size, percentage of the prevalence of each risk factor, and population size, and these factors are relatively stable over time as described next, the impact is very slight. First, the sample size here was sufficient as the sampling frame covered about 85% of older adults in China. Second, there was a slight percent change in the prevalence of each risk factor over a decade. For example, the prevalence of diabetes has increased from 9.7% in 2007 to 11.2% in 2017. Another limitation was that even though the sample size was considered relatively large, some risk factors identified in the study may not have a causal relationship with cognitive impairment. We used LASSO with cross-validation and previous experience to improve the possibility of the association between risk factors and the cognitive impairment. In addition, due to the crudeness in the measurement of predictors in the available data, the predictability of predictors and the performance of the model may be underestimated. For example, physical activity was measured by duration because the type, intensity, and frequency of the physical activity were not available in the data. The presence of such data granularity could enhance our model.

Our study had some strengths. First, we used nationally representative data representing 85% of Chinese older adults to develop the prediction model, and results could be generalized to older Chinese people. Second, we validated the prediction model using the method of temporal validation to enhance its practical use. Third, given the high mortality rates in older adults, we considered the competing risk of death to observe the precise effect of predictors on the outcome and the influence of death on the model. Finally, the predictors selected were easy to achieve and allowed health professionals in various care settings to quickly screen individuals with high risk of cognitive impairment.

## Data Availability Statement

The raw data supporting the conclusions of this article will be made available by the authors, without undue reservation.

## Ethics Statement

The studies involving human participants were reviewed and approved by Duke University and Peking University. Written informed consent to participate in this study was provided by the participants’ legal guardian/next of kin.

## Author Contributions

MH and YG conceived and designed the study. MH contributed to the statistical analysis and wrote the first draft of the manuscript. ZS contributed to the manuscript revision. TK, LX, and HF contributed to the writing of the final version of the manuscript. All authors contributed to the article and approved the submitted version.

## Conflict of Interest

The authors declare that the research was conducted in the absence of any commercial or financial relationships that could be construed as a potential conflict of interest.

## Publisher’s Note

All claims expressed in this article are solely those of the authors and do not necessarily represent those of their affiliated organizations, or those of the publisher, the editors and the reviewers. Any product that may be evaluated in this article, or claim that may be made by its manufacturer, is not guaranteed or endorsed by the publisher.

## Footnotes

1
https://opendata.pku.edu.cn/dataverse/CHADS

2
https://www.who.int/healthinfo/global_burden_disease/metrics_paf/en/
