# Supplementary material for: Derivation and Validation of the Cognitive Impairment Prediction Model in Older Adults: A National Cohort Study
Source: Front Aging Neurosci. 2022 Mar 4;14:755005. doi: 10.3389/fnagi.2022.755005 (PMC8931520; doi:10.3389/fnagi.2022.755005)
Supplement: Supplementary file 1 [file Data_Sheet_1.docx]

Table S1. Description of candidate predictors at baseline in the development dataset (n = 10,053)

| **Candidate predictors** | **Categories** | **N (%) or Median (IQRs)** |
| --- | --- | --- |
| **Demographic characteristics** | | |
| Age | - | 84.0 (75.0, 92.0) |
| Sex | Male | 4970 (49.4%) |
|  | Female | 5083 (50.6%) |
| Education | Illiteracy | 5723 (56.9%) |
|  | Primary school | 2540 (25.3%) |
|  | Secondary school | 1056 (10.5%) |
|  | High school | 428 (4.3%) |
|  | College | 306 (3.0%) |
| Marital status | Married and living with spouse | 3873 (38.5%) |
|  | Separated/divorced‎/never married‎ | 333 (3.3%) |
|  | Widowed | 5847 (58.2%) |
| Economic status | Rich | 1419 (14.1%) |
|  | So so | 6996 (69.6%) |
|  | Poor | 1638 (16.3) |
| **Lifestyles** |  |  |
| Smoking status | Never | 6307 (62.7%) |
|  | Former | 1668 (16.6%) |
|  | Current | 2078 (20.7%) |
| Smoking index | - | 0.0 (0.0, 270.0) |
| Drinking status | Never | 6661 (66.2%) |
|  | Former | 1398 (13.9%) |
|  | Current | 1994 (19.8%) |
| Drinking index (g/d) | 0 | 0.0 (0.0, 19.0) |
| Activity status | Never | 5870 (58.4%) |
|  | Former | 1097 (10.9%) |
|  | Current | 2078 (20.7%) |
| Activity duration (years) | - | 0 (0, 21.0) |
| Playing cards or mah-jongg | Almost everyday | 677 (6.7%) |
|  | Once a week | 464 (4.6%) |
|  | Once a month | 262 (2.6%) |
|  | Sometimes | 415 (4.1%) |
|  | Never | 8235 (81.9%) |
| Watching TV or listening to radio | Almost everyday | 5565 (55.4%) |
|  | Once a week | 1119 (11.1%) |
|  | Once a month | 500 (5.0%) |
|  | Sometimes | 534 (5.3%) |
|  | Never | 2335 (23.2%) |
| Doing garden work | Almost everyday | 947 (9.4%) |
|  | Once a week | 198 (2.0%) |
|  | Once a month | 126 (1.3%) |
|  | Sometimes | 228 (2.3%) |
|  | Never | 8554 (85.1%) |
| Taking part in some social activities | Almost everyday | 279 (2.8%) |
|  | Once a week | 177 (1.8%) |
|  | Once a month | 306 (3.0%) |
|  | Sometimes | 542 (5.4%) |
|  | Never | 8749 (87.0%) |
| Sleep quality | Good | 6698 (66.7%) |
|  | So so | 2399 (23.4%) |
|  | Bad | 956 (9.5%) |
| Sleep duration (h) | - | 8 (6, 9) |
| **Diseases** |  |  |
| Hypertension | Yes | 2166 (21.5%) |
|  | No | 7887 (78.5%) |
| Heart disease | Yes | 876 (8.7%) |
|  | No | 9177 (91.2%) |
| Diabetes | Yes | 257 (2.6%) |
|  | No | 9796 (97.4%) |
| Stroke or cardiovascular diseases | Yes | 542 (5.4%) |
|  | No | 9511 (94.6%) |
| **Physical function** |  |  |
| Body mass index | - | 20.2 (18.1, 22.7) |
| **Others** |  |  |
| Mood (feeling useless) | Always | 731 (7.3%) |
|  | Often | 1781 (17.7%) |
|  | Sometimes | 3537 (35.2%) |
|  | Seldom | 2151 (21.4%) |
|  | Never | 1853 (18.4%) |
| Systolic blood pressure | - | 133.5 (122.5, 149.0) |
| Diastolic blood pressure | - | 79.5 (72.0, 85.5) |


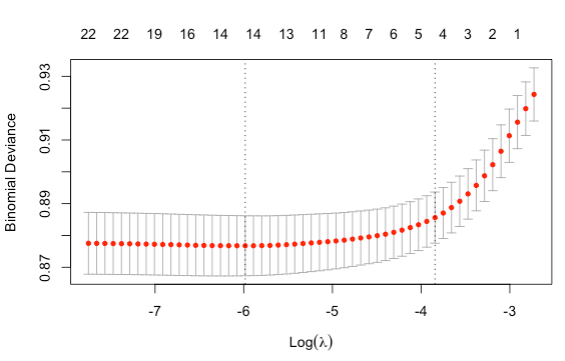
Figure S1. The number of predictors selection via LASSO with 10-fold cross validation.

Table S2. Predictors selected by LASSO with 10-fold cross validation.

| Number of predictors | Lambda values | Predictors selected |
| --- | --- | --- |
| 4 | Lambda (se) | Age, sex, education, and marital status |
| 15 | Minimal | Age, sex, education, activity duration, smoking index, drinking index, drinking status, sleep quality, economic status, marital status, hypertension, diabetes, stroke and cardiovascular diseases, playing cards or mah-jongg, watching TV or listening to radio |


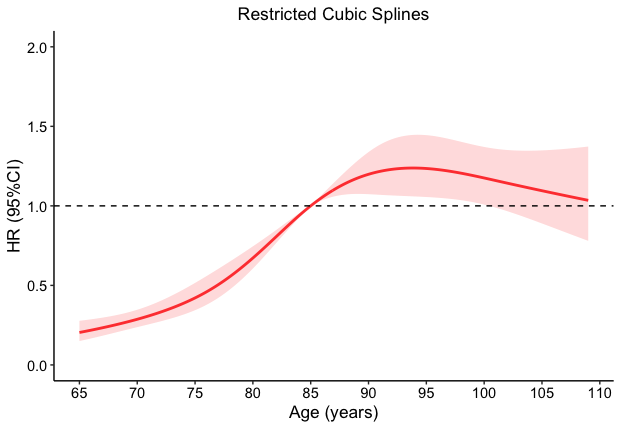
Figure S2. Nonlinear association between age and cognitive function using a model with 5 knots restricted cubic spline for age.

Curves show hazard ratio (HR) with 95% confidence interval (95%) compared with the chosen reference age of 85-year old.


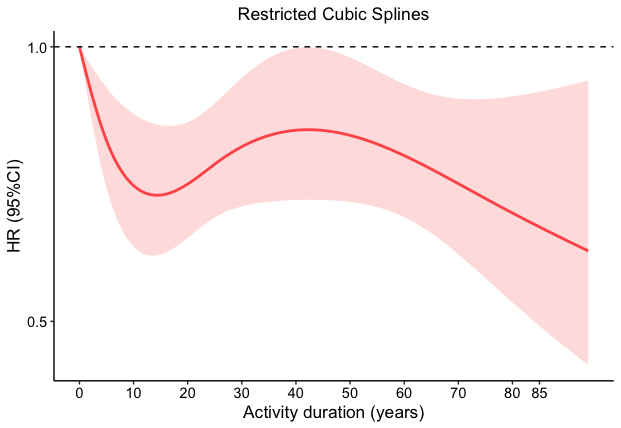


Figure S3. Nonlinear association between activity duration and cognitive function using a model with 4 knots restricted cubic spline for activity duration.

Curves show hazard ratio (HR) with 95% confidence interval (95%) compared with the chosen reference activity duration of zero.

Table S3. Descriptive characteristics of study population at baseline in development (n = 10,053) and validation cohort (n =)

| **Predictors** | **Categories** | **N (%) or Median (IQRs)** | | |
| --- | --- | --- | --- | --- |
|  |  | **Development cohort** |  | **Validation cohort** |
| Age | 65-74 | 2411 (24.0%) |  | 2302 (24.9%) |
|  | 75-84 | 2693 (26.8%) |  | 2509 (27.2%) |
|  | 85-94 | 3220 (32.0%) |  | 2573 (27.8%) |
|  | 95 and over | 1729 (17.2%) |  | 1856 (20.1%) |
| Sex | Male | 4970 (49.4%) |  | 4391 (47.5%) |
|  | Female | 5083 (50.6%) |  | 4849 (52.5%) |
| Education | Illiteracy | 5723 (56.9%) |  | 5327 (57.7) |
|  | Primary school | 2540 (25.3%) |  | 2514 (27.2%) |
|  | Secondary school | 1056 (10.5%) |  | 758 (8.2%) |
|  | High school | 428 (4.3%) |  | 326 (3.5%) |
|  | College | 306 (3.0%) |  | 315 (3.4%) |
| Marital status | Married and living with spouse | 3873 (38.5%) |  | 3197 (34.6%) |
|  | Separated/divorced‎/never married‎ | 333 (3.3%) |  | 367 (4.0%) |
|  | Widowed | 5847 (58.2%) |  | 5676 (61.4%) |
| Activity duration (years) | 0 | 5893 (58.6%) |  | 5486 (59.4%) |
|  | 1-15 | 1096 (10.9%) |  | 1319 (14.3%) |
|  | 16-40 | 1724 (17.1%) |  | 1216 (13.2%) |
|  | 41 and over | 1340 (13.3%) |  | 1219 (13.2%) |
| Playing cards or mah-jongg | Almost everyday | 677 (6.7%) |  | 559 (6.0%) |
|  | Once a week | 464 (4.6%) |  | 636 (6.9%) |
|  | Once a month | 262 (2.6%) |  | 279 (3.0%) |
|  | Sometimes | 415 (4.1%) |  | 320 (3.5%) |
|  | Never | 8235 (81.9%) |  | 7446 (80.6%) |
| Watching TV or listening to radio | Almost everyday | 5565 (55.4%) |  | 4203 (45.5%) |
|  | Once a week | 1119 (11.1%) |  | 1395 (15.1%) |
|  | Once a month | 500 (5.0%) |  | 423 (4.6%) |
|  | Sometimes | 534 (5.3%) |  | 553 (6.0%) |
|  | Never | 2335 (23.2%) |  | 2666 (28.9%) |
| Stroke or cardiovascular diseases | Yes | 542 (5.4%) |  | 429 (4.6%) |
|  | No | 9511 (94.6%) |  | 8811 (95.4%) |

Table S4. Multivariate-adjusted risk factors for cognitive impairment using Cox proportional hazard model and Fine-Gray model

| **Predictors** | Cox proportional hazard model | |  | Fine-Gray model | |
| --- | --- | --- | --- | --- | --- |
|  | Adjusted hazard ratio | 95% CI |  | Adjusted hazard ratio | 95% CI |
| Age at baseline |  |  |  |  |  |
| 65-74 | - | - |  | - | - |
| 75-84 | **2.46** | **2.04-2.97** |  | **2.13** | **1.77-2.56** |
| 85-94 | **4.34** | **3.60-5.24** |  | **3.02** | **2.51-3.64** |
| 95 and over | **4.52** | **3.68-5.57** |  | **2.63** | **2.15-3.23** |
| Sex |  |  |  |  |  |
| Male | - | - |  | - | - |
| Female | **1.52** | **1.36-1.71** |  | **1.60** | **1.43-1.78** |
| Education |  |  |  |  |  |
| Illiteracy | - | - |  | - | - |
| Primary school | **0.76** | **0.67-0.87** |  | **0.79** | **0.70-0.90** |
| Secondary school | **0.71** | **0.57-0.88** |  | **0.72** | **0.58-0.89** |
| High school | **0.68** | **0.48-0.95** |  | **0.69** | **0.50-0.96** |
| College | **0.67** | **0.47-0.96** |  | 0.77 | 0.55-1.08 |
| Marital status |  |  |  |  |  |
| Married and living with spouse | - | - |  | - | - |
| Widowed | 1.04 | 0.75-1.43 |  | 0.96 | 0.71-1.32 |
| Never married‎/divorced‎/separated | **1.15** | **1.01-1.30** |  | 1.09 | 0.96-1.23 |
| Activity duration (year) |  |  |  |  |  |
| 0 | - | - |  | - | - |
| 1-15 | 0.87 | 0.73-1.04 |  | 0.89 | 0.75-1.06 |
| 16-40 | 0.91 | 0.79-1.03 |  | 0.96 | 0.85-1.09 |
| 41 and over | **0.82** | **0.70-0.96** |  | **0.83** | **0.71-0.96** |
| Playing cards or mah-jongg |  |  |  |  |  |
| Almost everyday | - | - |  | - | - |
| Once a week | 1.23 | 0.88-1.72 |  | 1.20 | 0.88-1.65 |
| Once a month | 1.27 | 0.85-1.89 |  | 1.19 | 0.81-1.74 |
| Sometimes | 1.27 | 0.91-1.79 |  | 1.23 | 0.89-1.69 |
| Never | **1.34** | **1.06-1.68** |  | 1.19 | 0.96-1.48 |
| Watching TV or listening to radio |  |  |  |  |  |
| Almost everyday | - | - |  | - | - |
| Once a week | 1.06 | 0.90-1.24 |  | 1.01 | 0.87-1.18 |
| Once a month | 1.20 | 0.98-1.48 |  | 1.15 | 0.94-1.39 |
| Sometimes | 0.94 | 0.76-1.16 |  | 0.95 | 0.78-1.16 |
| Never | **1.14** | **1.03-1.29** |  | 1.09 | 0.98-1.22 |
| Stroke and cardiovascular diseases |  |  |  |  |  |
| Yes | - | - |  | - | - |
| No | **0.79** | **0.64-0.97** |  | 0.83 | 0.68-1.01 |

Note: Bold font highlights statistical significance.


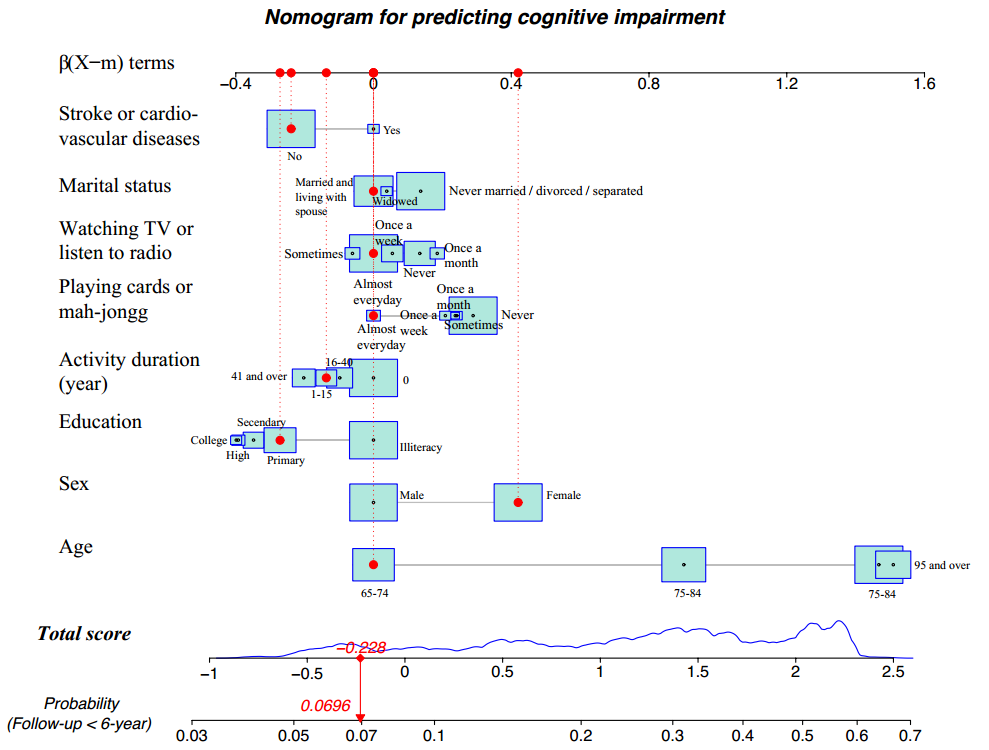
Figure S4. Nomogram for predicting cognitive impairment. For example, an older people (aged 65-74 years old; sex of female; education level of primary school; activity duration of 1-15 years; never playing cards or mah-jongg; watching TV or listen to radio almost every day; marital status of being married and living with spouse; and no stroke or cardiovascular diseases) had a total score of -0.228, corresponding to a probability of cognitive impairment of 6.96%

# Table S5. Reporting checklist for prediction model development/validation.

Reporting checklist for prediction model development/validation.

|  |  | Reporting Item | Page Number |
| --- | --- | --- | --- |
| **Title** |  |  |  |
|  | [#1](https://www.goodreports.org/reporting-checklists/tripod/info/#1) | Identify the study as developing and / or validating a multivariable prediction model, the target population, and the outcome to be predicted. | 1 |
| **Abstract** |  |  |  |
|  | [#2](https://www.goodreports.org/reporting-checklists/tripod/info/#2) | Provide a summary of objectives, study design, setting, participants, sample size, predictors, outcome, statistical analysis, results, and conclusions. | 4-5 |
| **Introduction** |  |  |  |
|  | [#3a](https://www.goodreports.org/reporting-checklists/tripod/info/#3a) | Explain the medical context (including whether diagnostic or prognostic) and rationale for developing or validating the multivariable prediction model, including references to existing models. | 6-8 |
|  | [#3b](https://www.goodreports.org/reporting-checklists/tripod/info/#3b) | Specify the objectives, including whether the study describes the development or validation of the model or both. | 8 |
| **Methods** |  |  |  |
| Source of data | [#4a](https://www.goodreports.org/reporting-checklists/tripod/info/#4a) | Describe the study design or source of data (e.g., randomized trial, cohort, or registry data), separately for the development and validation data sets, if applicable. | 8 |
| Source of data | [#4b](https://www.goodreports.org/reporting-checklists/tripod/info/#4b) | Specify the key study dates, including start of accrual; end of accrual; and, if applicable, end of follow-up. | 9 |
| Participants | [#5a](https://www.goodreports.org/reporting-checklists/tripod/info/#5a) | Specify key elements of the study setting (e.g., primary care, secondary care, general population) including number and location of centres. | 8 |
| Participants | [#5b](https://www.goodreports.org/reporting-checklists/tripod/info/#5b) | Describe eligibility criteria for participants. | 8-9 |
| Participants | [#5c](https://www.goodreports.org/reporting-checklists/tripod/info/#5c) | Give details of treatments received, if relevant | n/a |
| Outcome | [#6a](https://www.goodreports.org/reporting-checklists/tripod/info/#6a) | Clearly define the outcome that is predicted by the prediction model, including how and when assessed. | 9 |
| Outcome | [#6b](https://www.goodreports.org/reporting-checklists/tripod/info/#6b) | Report any actions to blind assessment of the outcome to be predicted. | n/a |
| Predictors | [#7a](https://www.goodreports.org/reporting-checklists/tripod/info/#7a) | Clearly define all predictors used in developing or validating the multivariable prediction model, including how and when they were measured | 10 |
| Predictors | [#7b](https://www.goodreports.org/reporting-checklists/tripod/info/#7b) | Report any actions to blind assessment of predictors for the outcome and other predictors. | n/a |
| Sample size | [#8](https://www.goodreports.org/reporting-checklists/tripod/info/#8) | Explain how the study size was arrived at. | n/a |
| Missing data | [#9](https://www.goodreports.org/reporting-checklists/tripod/info/#9) | Describe how missing data were handled (e.g., complete-case analysis, single imputation, multiple imputation) with details of any imputation method. | 10 |
| Statistical analysis methods | [#10a](https://www.goodreports.org/reporting-checklists/tripod/info/#10a) | If you are developing a prediction model describe how predictors were handled in the analyses. | 10-11 |
| Statistical analysis methods | [#10b](https://www.goodreports.org/reporting-checklists/tripod/info/#10b) | If you are developing a prediction model, specify type of model, all model-building procedures (including any predictor selection), and method for internal validation. | 10-11 |
| Statistical analysis methods | [#10c](https://www.goodreports.org/reporting-checklists/tripod/info/#10c) | If you are validating a prediction model, describe how the predictions were calculated. | 10-11 |
| Statistical analysis methods | [#10d](https://www.goodreports.org/reporting-checklists/tripod/info/#10d) | Specify all measures used to assess model performance and, if relevant, to compare multiple models. | 10-12 |
| Statistical analysis methods | [#10e](https://www.goodreports.org/reporting-checklists/tripod/info/#10e) | If you are validating a prediction model, describe any model updating (e.g., recalibration) arising from the validation, if done | 10-12 |
| Risk groups | [#11](https://www.goodreports.org/reporting-checklists/tripod/info/#11) | Provide details on how risk groups were created, if done. | 13 |
| Development vs. validation | [#12](https://www.goodreports.org/reporting-checklists/tripod/info/#12) | For validation, identify any differences from the development data in setting, eligibility criteria, outcome, and predictors. | 13 |
| **Results** |  |  |  |
| Participants | [#13a](https://www.goodreports.org/reporting-checklists/tripod/info/#13a) | Describe the flow of participants through the study, including the number of participants with and without the outcome and, if applicable, a summary of the follow-up time. A diagram may be helpful. | 13 |
| Participants | [#13b](https://www.goodreports.org/reporting-checklists/tripod/info/#13b) | Describe the characteristics of the participants (basic demographics, clinical features, available predictors), including the number of participants with missing data for predictors and outcome. | 13 |
| Participants | [#13c](https://www.goodreports.org/reporting-checklists/tripod/info/#13c) | For validation, show a comparison with the development data of the distribution of important variables (demographics, predictors and outcome). | 13 |
| Model development | [#14a](https://www.goodreports.org/reporting-checklists/tripod/info/#14a) | If developing a model, specify the number of participants and outcome events in each analysis. | 13 |
| Model development | [#14b](https://www.goodreports.org/reporting-checklists/tripod/info/#14b) | If developing a model, report the unadjusted association, if calculated between each candidate predictor and outcome. | 14 |
| Model specification | [#15a](https://www.goodreports.org/reporting-checklists/tripod/info/#15a) | If developing a model, present the full prediction model to allow predictions for individuals (i.e., all regression coefficients, and model intercept or baseline survival at a given time point). | 15 |
| Model specification | [#15b](https://www.goodreports.org/reporting-checklists/tripod/info/#15b) | If developing a prediction model, explain how to the use it. | 15 |
| Model performance | [#16](https://www.goodreports.org/reporting-checklists/tripod/info/#16) | Report performance measures (with CIs) for the prediction model. | 15 |
| Model-updating | [#17](https://www.goodreports.org/reporting-checklists/tripod/info/#17) | If validating a model, report the results from any model updating, if done (i.e., model specification, model performance). | n/a |
| **Discussion** |  |  |  |
| Limitations | [#18](https://www.goodreports.org/reporting-checklists/tripod/info/#18) | Discuss any limitations of the study (such as nonrepresentative sample, few events per predictor, missing data). | 23-24 |
| Interpretation | [#19a](https://www.goodreports.org/reporting-checklists/tripod/info/#19a) | For validation, discuss the results with reference to performance in the development data, and any other validation data | 17-23 |
| Interpretation | [#19b](https://www.goodreports.org/reporting-checklists/tripod/info/#19b) | Give an overall interpretation of the results, considering objectives, limitations, results from similar studies, and other relevant evidence. | 17-23 |
| Implications | [#20](https://www.goodreports.org/reporting-checklists/tripod/info/#20) | Discuss the potential clinical use of the model and implications for future research | 23 |
| **Other information** |  |  |  |
| Supplementary information | [#21](https://www.goodreports.org/reporting-checklists/tripod/info/#21) | Provide information about the availability of supplementary resources, such as study protocol, Web calculator, and data sets. | Supplementary material |
| Funding | [#22](https://www.goodreports.org/reporting-checklists/tripod/info/#22) | Give the source of funding and the role of the funders for the present study. | 2 |

None The TRIPOD checklist is distributed under the terms of the Creative Commons Attribution License CC-BY. This checklist can be completed online using <https://www.goodreports.org/>, a tool made by the [EQUATOR Network](https://www.equator-network.org) in collaboration with [Penelope.ai](https://www.penelope.ai)
